# Tensile and thermal properties of ceramic particulate and natural seaweed reinforced hybrid particulate polymer composites using the Taguchi approach

**DOI:** 10.1038/s41598-025-32130-7

**Published:** 2025-12-27

**Authors:** Ramraji Kirubakaran, Abilan Kumar, Harish Kumar Natchimuthu, Venkatachalam Gopalan, Kulasekharan Narasingamurthi

**Affiliations:** 1 School of Mechanical Engineering, Vellore Institute of Technology, Chennai, Tamilnadu 600127 India; 2 Centre for Advanced Materials and Innovative Technologies, Vellore Institute of Technology, Chennai, Tamilnadu 600127 India; 3 CAE Quality center, VinFast Automobiles, Long Bien distHaoi, Vietnam

**Keywords:** Seaweed filler, Ceramic fillers, Particulate composite, Tensile strength, Thermal conductivity, Taguchi analysis, Engineering, Materials science

## Abstract

Mechanical and thermal properties are critical when selecting materials for structural and semi structural applications. Mechanical strength ensures durability, while thermal conductivity is essential for performance under heat or thermal stress. Together, these properties help to develop composites suited for demanding fields like electronics, automotive and aerospace. This study investigates the effect of various weight percentages of natural and ceramic fillers on the mechanical and thermal behaviours of polymer composites (PCs). Epoxy composite laminates are fabricated using the hand layup method, incorporating seaweed (SW), silicon carbide (SiC) and aluminum oxide (Al₂O₃) as reinforcements. The design followed an L9 Taguchi orthogonal array, allowing for combinations of filler contents ranging from 2 to 6 wt.%. After fabrication, the laminates are subjected to waterjet cutting to prepare specimens for tensile and thermal conductivity testings. To identify the optimal parameter combinations, signal-to-noise (S/N) ratio analysis and analysis of variance (ANOVA) are employed. The results demonstrate that ceramic fillers significantly enhance both tensile strength (TS) and thermal conductivity (TC). The PCs of SWSCAO, containing 6 wt% Al₂O₃, exhibits maximum TS (87 MPa). TS of the SWSCAO3 composite is 22.84% greater than that of the SWSCAO1 composite with minimal ceramic filler (2 wt.% of SiC and Al₂O₃). This elucidates that composites with minimal amount of seaweed and maximum SiC and Al₂O₃ content are the most tensile effective. Specifically, the SWSCAO3 composite exhibited a 22.84% increase in TC compared to the SWSCAO1 composite, which contained the higher concentration of ceramic filler content (SiC and Al₂O₃). Thermal conductivity tests are performed at varying heat inputs (5W, 10W, 15W and 20W), with results showing conductivity values ranging from 1.91 to 2.71 W/m·K. Composites with higher Al₂O₃ content show improved thermal conductivity, whereas an increased proportion of seaweed filler tends to reduce it.

## Introduction

Particulate reinforced polymer composite (PRPC) materials are in high demand across industries like transportation, aerospace, packaging and construction, with natural particulate based composites playing a key role due to their environment friendly and cost efficiency^1,2^. Many studies explored reinforcement techniques using micro/nano particles from organic, inorganic and bio waste sources in polymer matrices^2,3^. Common ceramic reinforcements such as boron nitride (BN)^4^, silicon carbide (SiC)^5^, alumina^6^ boron carbide (B4C)^7^, carbon nanotubes^8^, Graphene^9^ and titanium diboride (TiB2)^10^ were frequently used in PCs. Moreover, these ceramic reinforcements in polymer matrix played crucial role in improving strength, ductility, toughness, thermal, electrical, morphological and tribological properties of PCs^3,11^. Epoxy resins, widely used as high toughness matrix materials, having low thermal conductivity^12^ was improved by incorporating metallic, carbon-based, or ceramic fillers. Metal and carbon fillers have higher thermal conductivity compared to ceramic fillers, but they also add more weight and increase the electrical conductivity of the composite. In contrast, ceramic fillers help to maintain lower weight and to provide electrical insulation property. Besides, ceramic fillers such as Al₂O₃, SiC and hBN can increase the thermal conductivity of polymer composites. Several investigations demonstrated that adding alumina (Al_2_O_3_) and silicon carbide (SiC) fillers greatly improves the mechanical, thermal and electrical resistance characteristics of PCs. These ceramic fillers’ intrinsic high electrical insulation and thermal conductivity were largely responsible for this improvement. Alumina offered robust electrical insulation and thermal stability, while silicon carbide’s superior thermal conductivity helped to improve heat dissipation. When used in combination, these fillers reduced thermal deterioration and enhanced the composite’s performance in hot and electrically demanding conditions^13–15^.

Kader et al.^16^ explored the epoxy/polysulfide copolymer, a highly flexible and resistant to various chemicals. Adding alumina powder to epoxy/polysulfide improved its mechanical properties, including TS, flexural strength (FS) and wear resistance. Optimal results were observed at 30% alumina, with further addition causing reductions in TS and FS due to particle agglomeration. Wear resistance was improved significantly, though dispersion issues at higher alumina levels limited performance gains. Bazrgari et al.^17^ investigated the performance of polymer nanocomposites reinforced with Al₂O₃ nanoparticles using ultrasonic mixing for uniform dispersion. Mechanical and tribological tests, including bending, impact and wear tests, showed that Al₂O₃ addition improved bending and impact strengths. Ozen et al.^18^ improved the thermal stability by incorporating surface modified Al_2_O_3_ nanoparticles into epoxy using the VARIM technique. Al_2_O_3_ was added at varying wt.% (1–5) after silane surface treatment. DSC, TGA and thermal conductivity tests revealed optimal thermal stability at 3 wt%. Rout et al.^5^ examined PCs reinforced with glass fibre and varying SiC filler content (0–40 wt.%). The study found that the inclusion of SiC enhanced thermal conductivity and stability, reaching a maximum thermal conductivity of 0.9906 W/m·K at 40 wt.% SiC.

Hybrid reinforcement fillers were also effective in boosting mechanical and thermal performances^19, 20^. Hoseini et al.^21^ used rice husk to synthesis with different wt.% of Al_2_O_3_/SiC composites using high temperature synthesis process. The study found that the inclusion both Al₂O₃/SiC fillers improved the mechanical strength and electrical characteristics of the PCs over the pure epoxy. Singh et al.^22^ investigated on me properties of glass fiber–Al₂O₃ particle–reinforced epoxy composites. Mechanical testing showed that increasing Al₂O₃ content improves properties, while larger particle sizes reduced performance. RSM and ANOVA were used to optimize and validate process parameters, confirming the results through testing. Sathish et al.^23^ studied the action of SiC and Al₂O₃ ceramic fillers on the mechanical response of FRP composites. The composites were compression molded with varying filler loadings. The optimal results were attributed to 8 wt% SiC and 20 wt% flax fiber which respectively led to the improved TS (44.56 MPa), FS (112.56 MPa) and IS (28.57 kJ/m^2^), indicating the reinforcement capacity of SiC. Ekpechi et al.^23,24^ explored the use of municipal solid waste, specifically coconut shell fibers, to create eco-friendly epoxy composites reinforced with SiC and Al₂O₃. Using hand lay-up and compression molding, they found that natural fiber-reinforced samples (S2–S4) significantly improved TS (up to 140.44 MPa) and TC (0.45 W/mK). Although pure epoxy (sample E) showed the highest IS (15.2 J/m^2^). Fan et al.^25^ investigated the thermal and mechanical performances of HDPE composites reinforced with hybrid fillers, namely Al₂O₃ particles and SiC whiskers, using maleated HDPE as a compatibilizer. The optimal filler ratio of Al₂O₃/SiC (1:4) achieved the highest TC (0.8876 W/m·K) and Young’s modulus (1160 MPa), while the 3:2 ratio offered improved thermal resistance (5.4 °C). The study confirmed the synergistic effect of hybrid fillers in enhancing HDPE composite properties over individual fillers.

Another bio waste reinforcement is seaweed. This ecofriendly reinforcement was used in polymer matrix to improve the mechanical strength and other structural properties^26,27^. Jumaidin et al.^27^ found that adding up to 40% seaweed waste improved the mechanical strength, thermal stability and biodegradability of sugar palm starch/agar PCs. SEM analysis revealed uniform fracture morphology, while TGA confirmed improved thermal resistance. The study concluded that such composites are suitable for short life applications like disposable trays and plates. Taguchi based techniques reduce the need for extensive trial and error testing by employing orthogonal arrays to systematically examine multiple factors and levels. This approach is particularly useful in the study of FRP composites, where production and testing can be both costly and time consuming. It is especially effective in identifying the most significant parameters affecting composite performance, such as fibre size, fibre content, matrix type, fibre chemical treatment, filler size and content^28–30^. Bera et al.^31^ optimized the wear behaviour of HDPE/SiC composites using the Taguchi method. They examined the effects of filler content, applied load and sliding velocity on wear rate and friction. Results showed that higher loads and velocities increased both wear rate and coefficient of friction. Arul et al.^32^ investigated Luffa acutangula fibre (LAF) reinforced PCs with varying wood dust content (0–30%). Using the Taguchi method, they identified optimal parameters for minimizing erosive wear (189.8 mg/kg), including 45 m/s velocity, 60° impingement angle and 20% wood dust. Mechanical properties were significantly improved at 20% wood dust loading, with tensile, flexural and impact strengths were increased by 17.56%, 48.78% and 54.64%, respectively.

Prior research indicated that ceramic and natural particulates significantly influence the performance of PCs, which have extensive applications in thermal, packaging, automotive and electrical fields. Ceramic fillers such as Al₂O₃ (alumina) and SiC (silicon carbide) exhibit high thermal conductivity, while seaweed serves as a cost effective, eco-friendly secondary reinforcement. Although many previous studies focused on single phase reinforcements to enhance mechanical and thermal properties, limited work has explored hybrid fillers combining ceramic and natural materials. Specifically, combinations of seaweed, Al₂O₃ and SiC in epoxy systems have not been extensively evaluated. This study aims to investigate the combined effect of ceramic fillers (Al₂O₃ and SiC) and seaweed filler in epoxy resin on tensile and thermal properties. The concentrations of different fillers (SW, Al₂O₃ and SiC) are optimised based on the Taguchi design (L9 orthogonal array).

## Materials and methods

### Materials

The materials used to fabricate the samples include epoxy resin (LY556) and hardener (HY951), along with Al₂O₃ (5 μm) and SiC (400 mesh size), all sourced from Herenba Instruments and Engineers, Chennai. Seaweed filler (< 75 μm), chosen for its sustainability and natural abundance, is supplied by AK Seaweeds, Ramanathapuram, India. A 270 × 270 × 5 mm^3^ mould is prepared with silicone rubber to make the laminates.

### Taguchi design and composite preparation

**Fig. 1:**
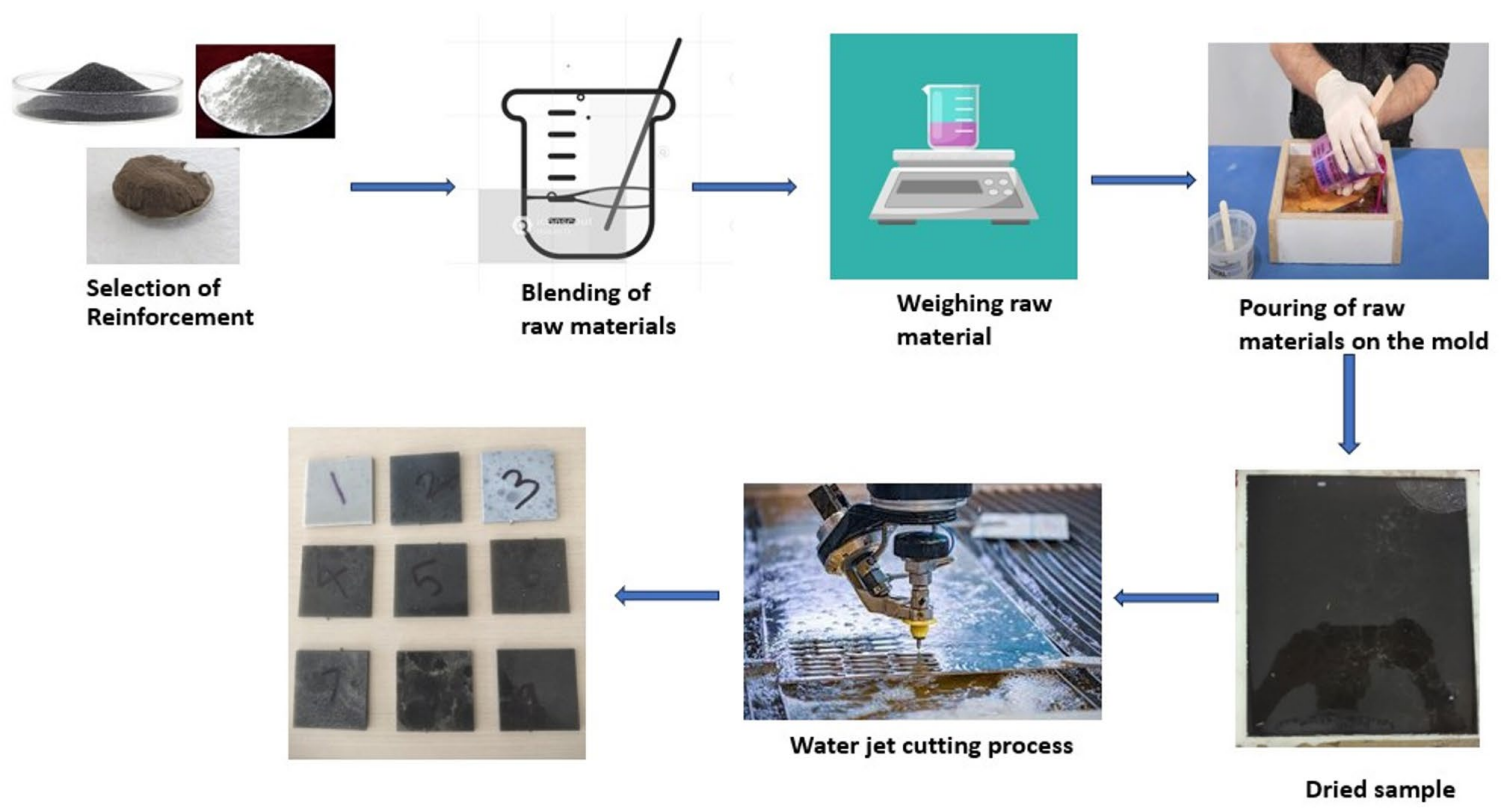
Fabrication process of SWSCAO polymer composite.

The hand layup process is used to fabricate the selected three phase hybrid particulate reinforced polymer (HPRP) composites due to its suitability for small-batch production, flexibility, cost-effectiveness and minimal equipment requirements. Additionally, its low spatial and equipment demands make it ideal for small scale businesses^33^. Table 1 presents the factors (SW, SiC and Al_2_O_3_) and their respective levels by wt.%. Table 2 presents the Taguchi L9 array generated using Minitab 19, which efficiently evaluates the effects of multiple factors at three levels with a significantly reduced number of experimental trials. This design offers systematic optimization, cost savings, robustness and improved data analysis capability. The Taguchi L9 array is employed for the fabrication of seaweed/silicon carbide/aluminium oxide (SWSCAO) polymer composites, incorporating various levels of seaweed, SiC, and Al₂O₃ fillers (2–6 wt.%). To make it simple to remove the SWSCAO laminate, a release agent is sprayed on the mould inside surface. A mechanical stirrer is employed to achieve homogeneous mixing of the three-phase ceramic and natural filler reinforcements within the epoxy matrix. The mixture of epoxy and fillers with hardener (10:1 ratio) is evenly poured inside the mold and hybrid particulate polymer laminates are allowed to cure at room temperature for 24 h followed by a longer ambient conditioning period, then the sample is removed from the mold. After fabrication process, the samples undergo abrasive water jet (AWJ) cutting process to cut the samples into different pieces for different testings. The potential for moisture absorption during the waterjet cutting process is taken into account. To minimize variations caused by absorbed moisture, all samples are dried in a hot oven at 70 °C for 24 h prior to conduct the mechanical and thermal testings. The SWSCAO hybrid PCs manufacturing process is depicted in Fig. 1.

**Table 1:** Process parameters (SW, SiC and Al_2_O_3_) and their levels.

Parameters	Wt.%		
	Level 1	Level 2	Level 3
SW	2	4	6
SiC	2	4	6
Al_2_O_3_	2	4	6

**Table 2:** Experimental Taguchi L9 array used for the fabrication of SWSCAO composites.

S.no	Composite Designation	Seaweed filler (wt.%)	SiC filler (wt.%)	Al_2_O_3_ filler (wt.%)
1	SWSCAO1	2	2	2
2	SWSCAO2	2	4	4
3	SWSCAO3	2	6	6
4	SWSCAO4	4	2	4
5	SWSCAO5	4	4	6
6	SWSCAO6	4	6	2
7	SWSCAO7	6	2	6
8	SWSCAO8	6	4	2
9	SWSCAO9	6	6	4

### Tensile test

**Fig. 2:**
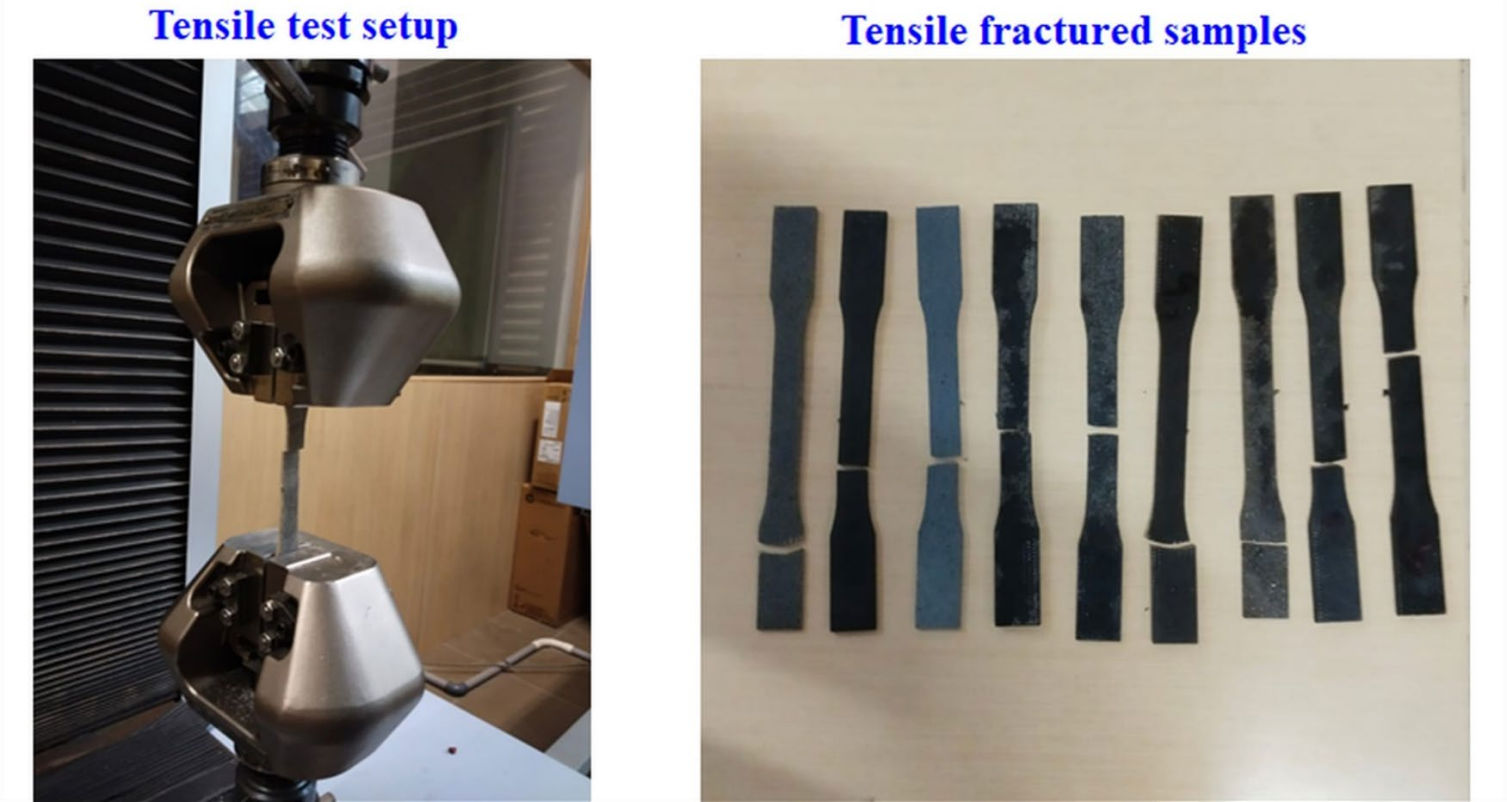
Tensile test setup and corresponding SWSCAO polymer composite samples.

Tensile testing of the SWSCAO polymer composite samples (dog bone shape) is carried out using a Universal Testing Machine AG-Xplus 50 KN (Make-Shimadzu and measurement accuracy: 0.001 N) to evaluate their tensile strength under uniaxial tension at a strain rate of 3 mm/min (ASTM D638 standard). The tensile test setup and corresponding SWSCAO composite samples are shown in Fig. 2.

### Thermal conductivity test

**Fig. 3:**
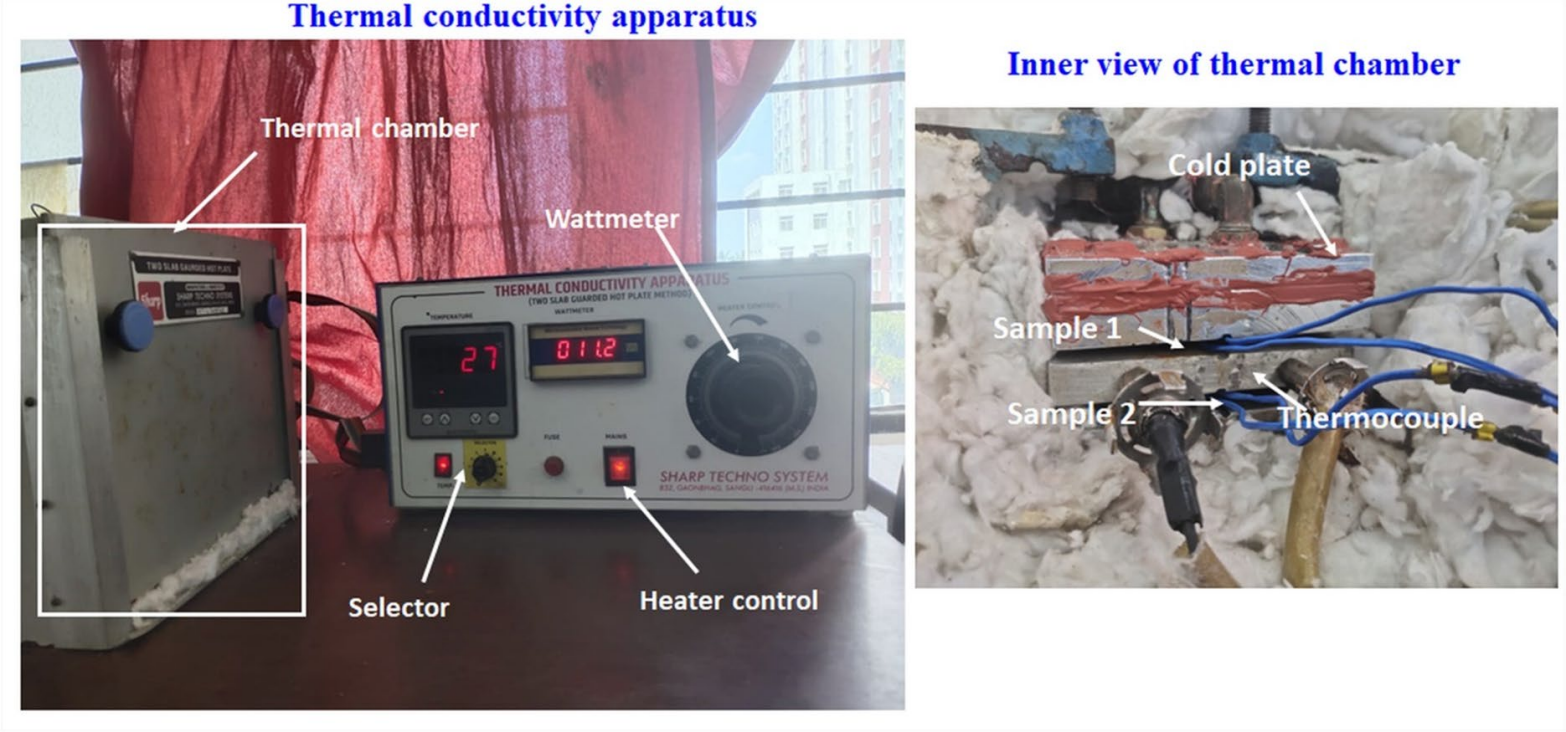
Thermal conductivity test setup for the SWSCAO polymer composite samples.

The TC of the SWSCAO polymer composite samples is measured using two slabs TC apparatus (Make: Sharp Techno Systems and hot plate temperature range: Ambient to 150 °C). For measuring thermal conductivity, 2 identical samples are cut by AWJ cutting with a size 50 × 50 × 10 mm^3^^34^. The apparatus consists of two slabs and the sample is placed inside the slabs by sticking the samples with the wire using an insulation tape. After this arrangement, the water flowing pipe and the apparatus is turned on and set the voltage of 5 Watts. The temperature in the T1, T2, T3, T4 regions are noted for every 15 min for a time period of 2 h. After that the apparatus is turned off and it takes around three hours for the apparatus to cool down. The similar procedure is followed for 10, 15 and 20 Watts. Figure 3 shows the thermal conductivity test setup for the SWSCAO PCs samples. The TC of the SWSCAO polymer composite samples is calculated by Eq. (1).

1 $$K = \frac{Q*\Delta X}{{A*\Delta T}}$$

where K-TC (W/m.K), Q is the heat flow rate (W), Δx- conducting surface thickness (mm), A-conducting area (mm^2^), ΔT-temperature difference (°C).

## Results and discussions

### Tensile strength

**Fig. 4:**
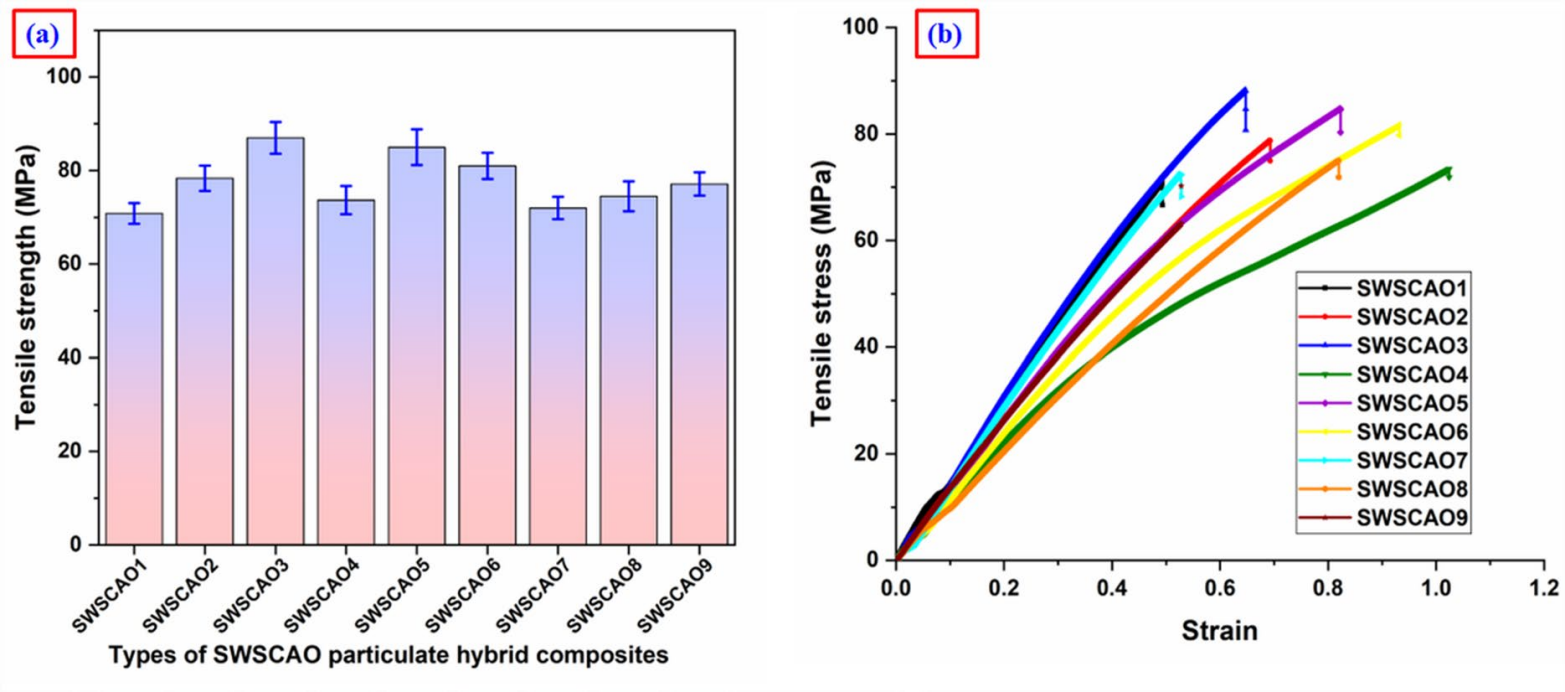
(**a**) TS and (**b**) Stress strain of SWSCAO polymer composites.

Figure 4a illustrates the TS of various SWSCAO particulate filled polymer composites. The incorporation of ceramic fillers into the epoxy resin improves the TS of these SWSCAO composites. Among them, the SWSCAO composite with 6 wt% Al₂O₃ exhibited the highest TS (87 MPa). According to the graph, the TS of the SWSCAO3 composite is 22.84% higher than that of the SWSCAO1 composite, which contains the lowest ceramic filler content (2 wt.% of SiC and Al₂O₃). These results indicate that composites with minimal seaweed content and maximum SiC and Al₂O₃ loading deliver the best tensile performance. The improvement in TS is attributed to the uniform dispersion of ceramic fillers, which enhances the material’s ability to bear tensile loads. Ceramic fillers improve TS by occupying micro voids within the matrix, minimizing pore formation and restricting polymer chain mobility during loading^3^. In contrast, higher concentrations of the natural seaweed filler tend to reduce the TS of the SWSCAO polymer composite. The addition of SW to the composites improves elongation before fracture compared to the SWSCAO composite, as illustrated by the typical stress–strain curve in Fig. 4b. This indicates that the stiffness and toughness of the material are enhanced due to the epoxy matrix reinforced with ceramic fillers. When these fillers are present in optimal concentrations, the material can endure greater strain before failure, which is attributed to improved crack bridging ability and more effective stress transfer^2^.

**Fig. 5:**
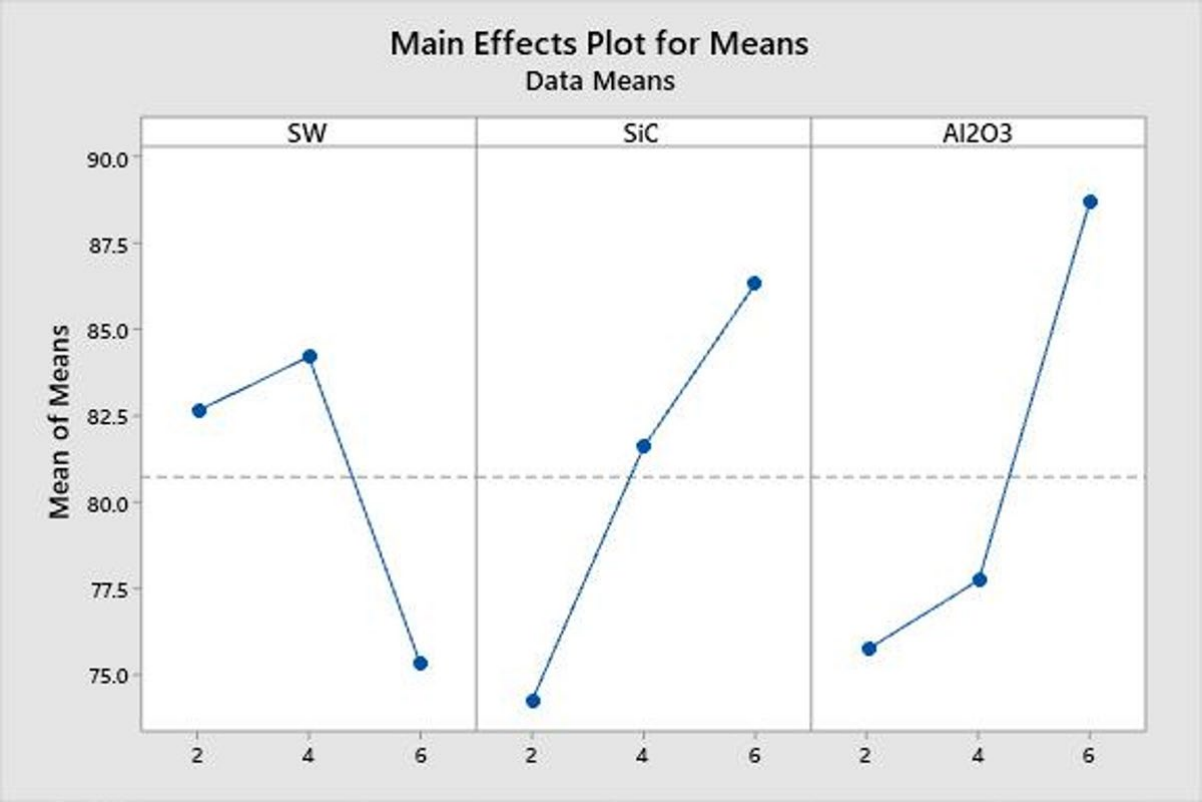
TS main effect plot for means.

Table 3 present the both response tables (means and signal-to-noise ratio), which further confirm that the SiC filler has the most significant effect on the tensile strength of the SWSCAO polymer composites. This is evident from its highest ranking in both the response means and S/N ratio. The second most influential factor is Al₂O₃, which contributes positively to the TS. Hence, optimizing the content of these ceramic fillers is essential for enhancing the mechanical performance of hybrid filler-based PCs^31^. In contrast, a decline in TS is observed when the seaweed (SW) filler content exceeds 4 wt.%, likely due to poor SW and epoxy resin adhesion, as well as the creation of voids^3, 34, 35^. Figure 5 shows the TS main effect plot for means. Among the three variables, SW filler appears to have the least impact on TS. This aligns with previous studies, which suggest that the non-uniform dispersion of organic natural fillers like SW can reduce reinforcement efficiency^27^.

**Table 3:** Means and SN ratio response table.

Level	Response table					
	Means			SN ratio		
	SW	SiC	Al_2_O_3_	SW	SiC	Al_2_O_3_
1	78.73	72.16	75.44	37.89	37.17	37.54
2	79.89	79.29	76.39	38.03	37.97	37.66
3	74.54	81.71	81.33	37.44	38.23	38.18
Delta	5.35	9.54	5.89	0.59	1.07	0.64
Rank	3	1	2	3	1	2

The experimental findings are statistically analyzed using ANOVA^29,36^ to evaluate the influence of key parameters namely, the weight percentages of SiC and Al₂O₃ fillers—on the TS of the fabricated SWSCAO composite samples. ANOVA helps to determine the significance of each independent variable, as indicated by the *P*-values listed in Table 4. A *P*-value less than 0.005 indicates statistical significance^27,28^. In this study, the incorporation of SiC filler demonstrated a highly significant impact on the t TS of the SWSCAO composite, as indicated by an F-value of 60.76 and a *P*-value of 0.016 obtained from the ANOVA analysis. These statistical values suggest that SiC plays a critical role in influencing the observed variation in TS, with the low *P*-value confirming its statistical significance at a 95% confidence level. Furthermore, this result is strongly supported by the high coefficient of determination (R^2^ = 99.06%), indicating that the developed model effectively accounts for nearly all the variability in the experimental data. This high R^2^ value reinforces the reliability and predictive capability of the model in explaining the influence of SiC on the mechanical performance of the SWSCAO composite. In contrast, the seaweed (SW) filler demonstrates a minimal statistical impact with a *P*-value of 0.049, indicating it has a relatively minor influence. Furthermore, the low adjusted mean square (Adj MS = 1.215) for the error term enhances the credibility of the ANOVA results by reducing unexplained variation. Table 5 provides a detailed comparison between the experimentally obtained tensile strength values of SWSCAO polymer composite and those predicted using the regression Eq. (2).

2 $$\begin{aligned} {\text{TS }}\left( {{\mathrm{MPa}}} \right) = & {77}.{72}0 + \left( {{1}.0{1}0 *{\mathrm{SW2}}} \right) + \left( {{2}.{17}0 *{\mathrm{SW4}}} \right){-} \left( {{3}.{18}0 *{\mathrm{SW6}}} \right) \\ & {-} \left( {{5}.{557}*{\mathrm{SiC2}}} \right) + \left( {{1}.{57}0*{\mathrm{SiC4}}} \right) + \left( {{3}.{987}*{\mathrm{SiC6}}} \right) \\ & - \left( {{2}.{28}0*{\mathrm{Al}}_{{2}} {\mathrm{O}}_{{3}} {2}} \right) - \left( {{1}.{333}*{\mathrm{Al2O3}}*{4}} \right) + \left( {{3}.{613}*{\mathrm{Al}}_{{2}} {\mathrm{O}}_{{3}} {6}} \right) \\ \end{aligned}$$

**Table 4:** ANOVA results for TS of SWSCAO particulate polymer composites.

Source	DF	Adj SS	Adj MS	F-value	*P*-value
SW	2	47.524	23.762	19.55	0.049
SiC	2	147.705	73.852	60.76	0.016
Al_2_O_3_	2	60.097	30.049	24.72	0.039
Error	2	2.431	1.215		
Total	8	257.757			
S = 0.0491031	R^2^ = 99.06%	R-sq.(adj) = 96.23%	R-sq.(pre) = 80.9%

**Table 5:** TS value comparison between regression equation predicted and experimental results.

Composite designation	TS (MPa)	Regression	Error (%)
SWSCAO1	70.82	70.9	− 0.12
SWSCAO2	78.37	78.97	− 0.77
SWSCAO3	87	86.33	0.78
SWSCAO4	73.67	73	0.91
SWSCAO5	85	85.08	− 0.1
SWSCAO6	81	81.6	− 0.75
SWSCAO7	72	72.6	− 0.84
SWSCAO8	74.5	73.83	0.9
SWSCAO9	77.12	77.2	− 0.11

**Fig. 6:**
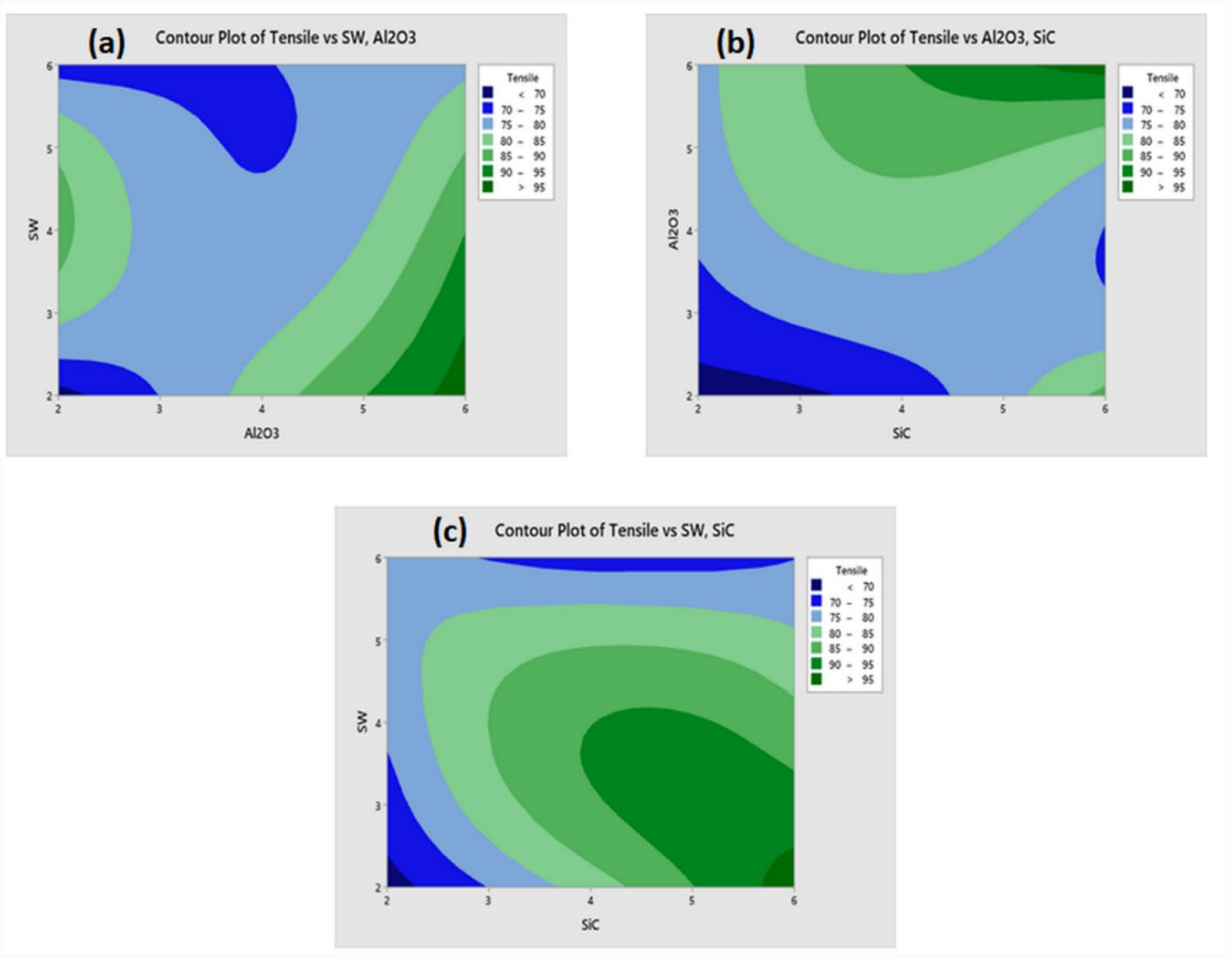
TS contour plot of (**a**) SW/ Al_2_O_3_ (**b**) Al_2_O_3_/SiC and (**c**) SW/SiC.

The relationship between two independent variables and a dependent variable is depicted by a contour plot on a single graph. The two independent variables are represented by the X and Y axes and the dependent variable is represented by the contour lines and band. It looks for X and Y pairings that yield advantageous results^36^. Figure 6a–c, the contour plot depicting TS analysis, indicates that, between various fillers weight percentage, weight percentage has a minimal impact on TS when combined with higher concentration of SW. The contour plot of TS as a function of seaweed (SW) and Al₂O₃ content is illustrated in Fig. 6a. This contour map effectively demonstrates the interaction between these two fillers, revealing that while SW does influence the TS, Al₂O₃ exhibits a more prominent and consistent effect in enhancing the TS of the composite. Figure 6b presents the contour surface for SiC and Al₂O₃, which highlights that both ceramic fillers significantly contribute to improvements in TS, with noticeable synergy between them. Likewise, the contour map depicting the interaction between SW and SiC suggests that although SW plays a role in modifying TS, the influence of SiC is more dominant (Fig. 6c). These plots collectively indicate that among the three fillers studied, the ceramic reinforcements (SiC and Al₂O₃) are more critical in improving the TS of the composite material^2,16^.

**Fig. 7:**
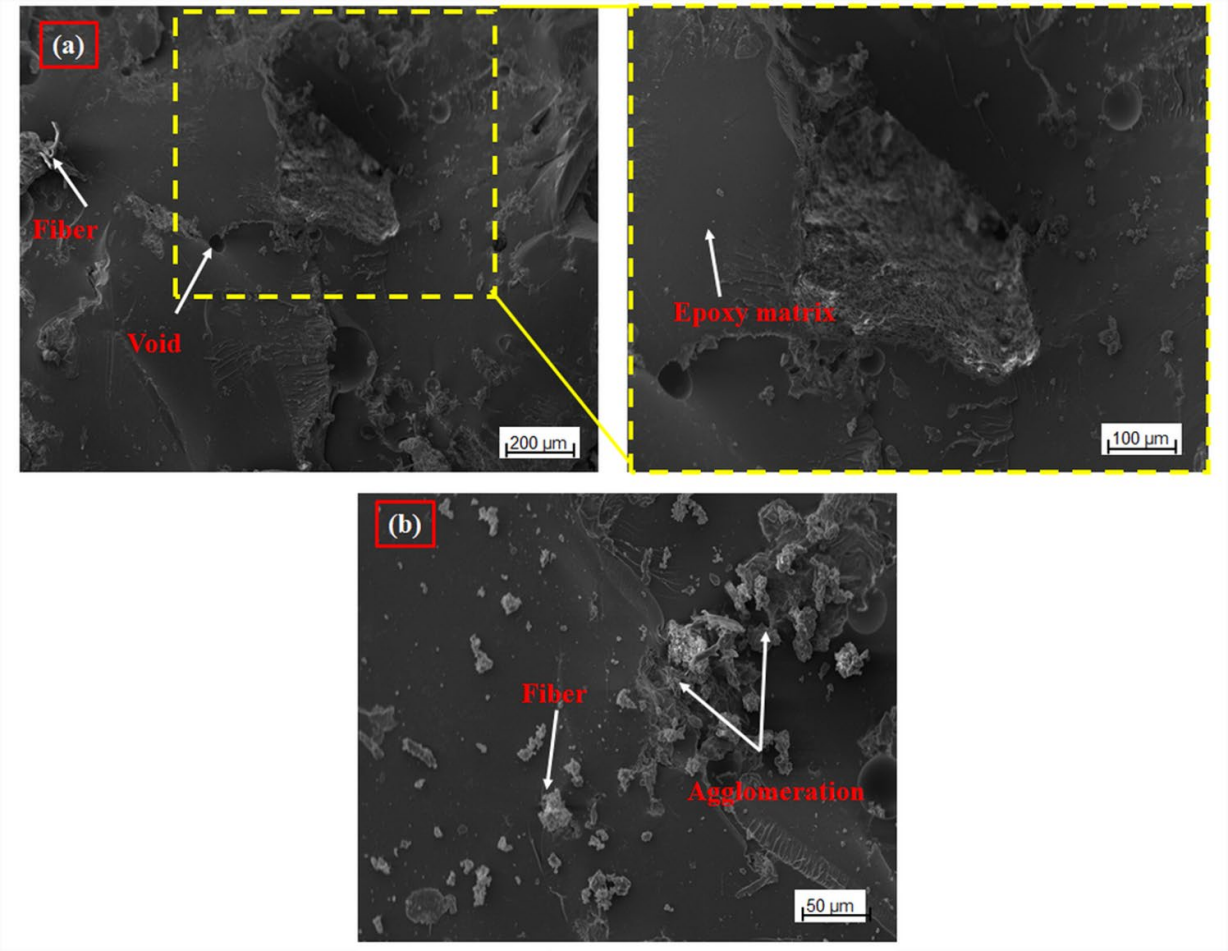
Morphology surface of SWSCAO polymer composites.

Figure 7 shows the microstructural dispersion of seaweed/ceramic interlaced hybrid epoxy composites for Samples SWSCAO3 and SWSCAO7, as seen through SEM images. The images indicate a reasonably consistent dispersion of the hybrid particulate reinforcements, which consists of both seaweed fiber and ceramic particulates (Al_2_O_3_/SiC), within the epoxy matrix. The dispersion uniformity is most evident in the SWSCAO3 composite with the lowest filler loading (Fig. 7a), with the particles being fully integrated without significant agglomeration. Figure 7b related to Sample 7, with a higher total filler percentage, shows noticeable agglomeration. The agglomeration is due to the high amount and surface area of the three phase fillers, leading to particle interactions that undermine the dispersion^5,11^. Agglomeration can be seen as stress concentrators which deteriorate the loaded qualities of the SWSCAO composite. The extent of this microstructural change has a major influence on the TS of the composites as seen from their stress strain curves. The composite with the low seaweed and high ceramic particulate has better tensile strength. The epoxy matrix has better ability to wet and encapsulate of the filler particles for good load transfer while minimizing voids or crack initiation points.

### Thermal conductivity (TC)

**Fig. 8:**
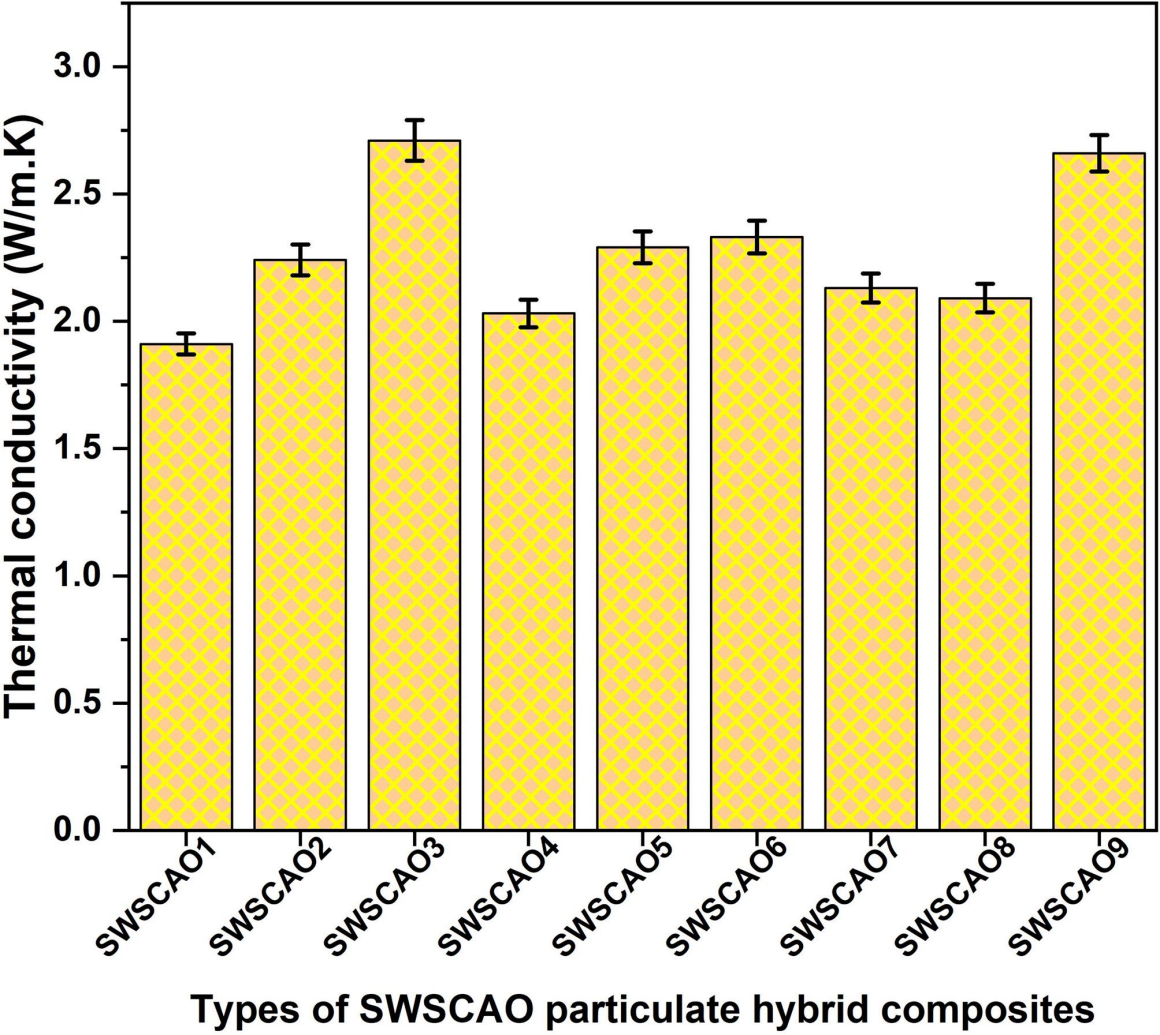
TC of SWSCAO polymer composites.

The TC test results for the nine SWSCAO composite samples are presented in Fig. 8. The TC values range from 1.91 to 2.71 W/m K. The SWSCAO composite containing 2 wt.% seaweed along with Al₂O₃ and SiC exhibited the lowest TC value of 1.91 W/m·K. In contrast, the composite with 6 wt.% SiC, combined with Al₂O₃ and seaweed, demonstrated the highest TC value of 2.71 W/m·K. These findings suggest that the TC of the composites improves with increasing weight percentages of Al₂O₃ and SiC fillers. This enhancement is likely due to the inherently high TC of these ceramic materials, which facilitate more efficient heat transfer through the composite matrix.

**Fig. 9:**
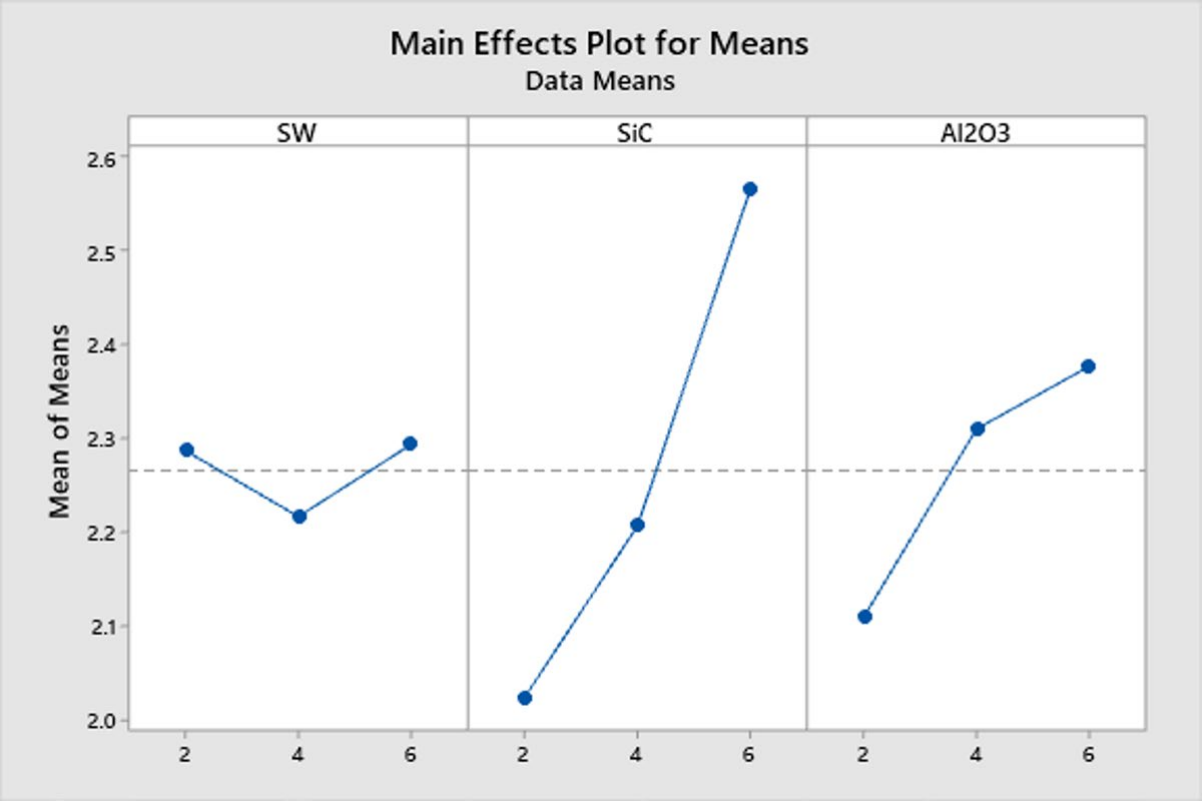
TC main effect plot for mean.

Table 6 present the response tables for both the means and signal-to-noise (S/N) ratio, which further confirm that the Al_2_O_3_ filler has the most significant effect on the TC of the SWSCAO polymer composites. Response table showing the effect of three different factors (Seaweed, SiC and Al_2_O_3_) on a response variable across three levels. The values in each column represent the mean response at each level for the respective factor. The ‘Delta’ row represents the influence of each factor on the response by showing the difference between the highest and lowest mean response values. A higher Delta value suggests a greater impact, leading to the ranking of factors in order of significance. Based on this ranking, Al_2_O_3_ has the highest influence, followed by SiC and then Seaweed. The Main Effects Plot for means illustrates the factors SW, SiC and Al_2_O_3_ influencing the response variable (Fig. 9). The SW factor shows a fluctuating but relatively stable trend, indicating a minor effect on the response. In contrast, Silicon carbide exhibits a strong upward trend, suggesting a significant positive impact as its levels increase. Al_2_O_3_ also shows a noticeable increasing trend, indicating its contribution to the response variable. The steeper slopes of SiC and Al_2_O_3_ suggest that these factors have a more substantial effect, while SW appears to have a weaker influence.

**Table 6:** Means and S/n ratio response table.

Level	Response table					
	Means			S/n ratio		
	SW	SiC	Al_2_O_3_	SW	SiC	Al_2_O_3_
1	2.287	2.023	2.110	38.25	37.40	37.54
2	2.217	2.207	2.310	38.48	38.20	37.80
3	2.293	2.567	2.377	37.53	38.67	38.92
Delta	0.077	0.543	0.267	0.95	1.28	1.39
Rank	3	2	1	3	2	1

Table 7 shows the ANOVA table analysing the effects of SW, SiC and Al_2_O_3_ on the response variable. The SiC factor exhibits the most significant effect, with a high F-value (95.06) and a *P*-value of 0.010, indicating a strong influence. Al_2_O_3_ also has a significant effect (F = 23.96, *P* = 0.040), suggesting it contributes notably to variance. However, SW does not show statistical significance (*P* = 0.08), meaning its impact is minimal. The error variance (Adj MS = 0.002411) is low, which enhances the reliability of the results. The total sum of squares (0.589622) confirms that the majority of variance is attributed to the significant factors, primarily SiC and Al_2_O_3_. Overall, the model successfully identifies key influential factors while minimizing error. The ideal TC of the SWSCAO polymer composite, taking into account the cumulative influence of the aforementioned parameters, is predicted to be around 3.56 (W/m.K), according to regression Eq. (3). Based on the regression Eq. (3), the predicted and experimental results are calculated and presented in Table 8.

3 $$\begin{aligned} {\mathrm{TC}} = & \left( {{\mathrm{W}}/{\mathrm{m}}.{\mathrm{K}}} \right){2}.{2656} + \left( { 0.0{211}*{\mathrm{SW2}}} \right){-} \left( {0.0{489}*{\mathrm{SW4}}} \right) + \left( {0.0{278}*{\mathrm{SW6}}} \right) \\ & {-} \left( {0.{2422}*{\mathrm{SiC2}}} \right) - \left( {0.0{589}*{\mathrm{SiC4}}} \right) + \left( {0.{3}0{11}*{\mathrm{SiC6}}} \right) \\ & - \left( {0.{1556}*{\mathrm{Al}}_{{2}} {\mathrm{O}}_{{3}} {2}} \right) + \left( {0.0{444}*{\mathrm{Al}}_{{2}} {\mathrm{O}}_{{3}} {4}} \right) \, + \left( {0.{1111}*{\mathrm{Al}}_{{2}} {\mathrm{O}}_{{3}} {6}} \right) \\ \end{aligned}$$

**Table 7:** ANOVA results for particulate composites.

Source	DF	Adj SS	Adj MS	F-value	*P*-value
SW	2	0.010822	0.005411	2.24	0.08
SiC	2	0.458422	0.229211	95.06	0.010
Al_2_O_3_	2	0.115556	0.057778	23.96	0.040
Error	2	0.004822	0.002411		
Total	8	0.589622			
S = 0.0491031	SR-sq. = 99.18%	R-sq.(adj) = 96.73%		R-sq.(pred) = 83.44%

**Table 8:** TC value comparison between regression equation predicted and experimental results.

Composite designation	TC W/m K	Regression	Error (%)
SWSCAO1	1.91	1.889	1.1
SWSCAO2	2.24	2.273	− 1.48
SWSCAO3	2.71	2.699	0.41
SWSCAO4	2.03	2.019	0.55
SWSCAO5	2.29	2.269	0.92
SWSCAO6	2.33	2.363	− 1.42
SWSCAO7	2.13	2.163	− 1.55
SWSCAO8	2.09	2.079	0.53
SWSCAO9	2.66	2.639	0.79

**Fig. 10:**
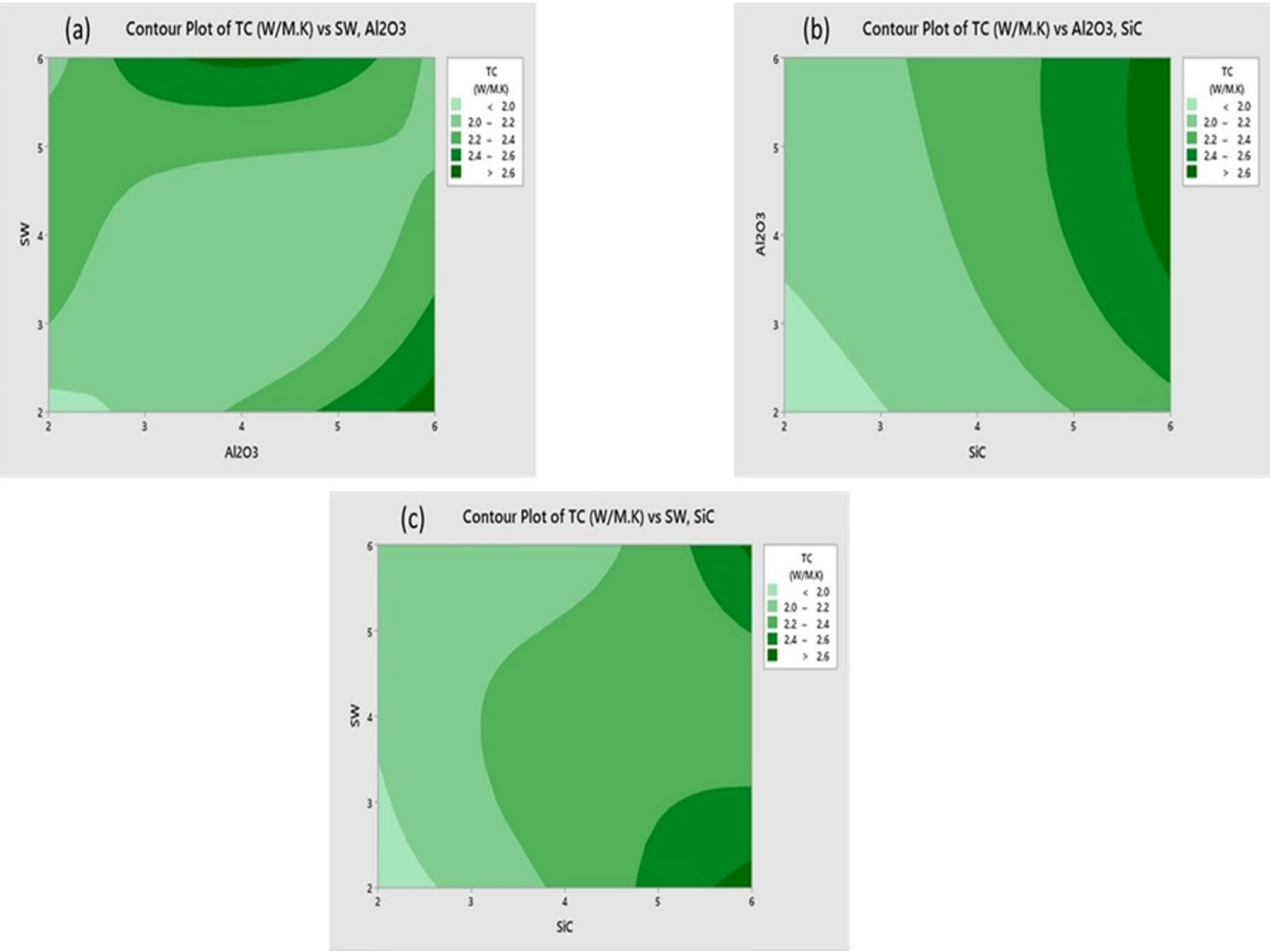
TC contour plot of (**a**) SW/ Al_2_O_3_ (**b**) Al_2_O_3_/SiC and (**c**) SW/SiC.

A contour plot is a two-dimensional graphical representation used to visualize three-dimensional data through colour gradients. In this context, three variables such as SW, SiC (silicon carbide) and Al₂O₃ are analysed. Two of these variables (e.g., SW and SiC or SW and Al₂O₃) are plotted on the X and Y axes, while the third variable, thermal conductivity (TC), is represented by colour gradients (Fig. 10a–c). Each colour region on the plot corresponds to a constant TC value, allowing trends, gradients and correlations among variables to be examined easily^35^. Figure 10a illustrates the thermal conductivity (TC) in W/m.K as a function of Al₂O₃ and SW contents. The colour shades range from pale green (representing low TC values) to dark green (representing high TC values). The legend, located on the right, categorizes TC into five ranges: below 2.0, 2.0–2.2, 2.2–2.4, 2.4–2.6 and above 2.6 W/m·K. The contour lines interconnect regions of equal TC values, enabling visual identification of patterns and optimal combinations. This helps in understanding the response of thermal conductivity to variations in Al₂O₃ and SW levels. Another contour plot presents TC as a function of SiC and Al₂O₃ contents (Fig. 9b). Similar to the previous case, darker green shades indicate higher TC values. The visual trends suggest that combinations with higher concentrations of SiC and Al₂O₃ result in enhanced TC, with the darkest green shades appearing in the upper regions of the plot. A third contour plot shows TC as a function of SiC and SW contents (Fig. 10c). Again, TC values are represented by shades of green, with darker shades indicating higher TC. The darkest regions appear in the upper right-hand corner, where SiC content is highest. The pattern of the contour lines suggests a nonlinear relationship, indicating that both SiC and SW significantly influence TC, but their effects are not purely additive or linear. Overall, these contour plots clearly demonstrate the synergistic and complex interactions between ceramic (SiC, Al₂O₃) and natural (SW) fillers in influencing the thermal conductivity of the composite material.

## Conclusions

In this work, based on Taguchi method, the SWSCAO polymer composites are fabricated by using hand layup process. Combined effect of natural seaweed filler and SiC/Al_2_O_3_ ceramic filler on tensile and thermal properties are studied. The following major observations are made from the results.

1. The optimal configuration for achieving superior TS in the SWSCAO particulate polymer composite is identified as a weight fraction of 6% SiC, 2% Al₂O₃ and 2% seaweed.
2. Among the three factors considered in this study, SiC loading is the most significant in determining TS. The composite containing 6 wt.% SiC has a TS of 87 MPa, which is greater than any of the other composites.
3. Thermal conductivity test is taken and the results show that the sample with higher weight percentage of silicon carbide has higher thermal conductivity. The ANOVA table analyses the effects of Al_2_O_3_, SiC and SW on the response variables.
4. The contour plots reveal the combined influence of two factors (SiC and Al₂O₃) and their contribution to the overall TS and TC. The contour map illustrating the interaction between SW and SiC indicates that SiC has more influence on TS. Similarly, in the interaction between SW and Al₂O₃, although SW has some effect on TC, Al₂O₃ is the more influential factor.
5. Though the primary objective of this research is to study TS and thermal conductivity for potential industrial applications, it is also important to broaden the assessment to include other critical properties such as hardness, flexural strength, impact strength, dynamic mechanical, compressive strength, electrical conductivity and acoustic behaviour.

## Data Availability

The datasets used and/or analysed during the current study available from the corresponding author on reasonable request.
